# Anti-Inflammatory Effects of Progesterone on Human Microglia via TLR4/NLRP3 Pathway Modulation: Relevance to Drug-Resistant Epilepsy

**DOI:** 10.3390/ph19060920

**Published:** 2026-06-11

**Authors:** Ramona Meanti, Maria Laura Criscione, Emma Sartori, Laura Rizzi, Elena Bresciani, Mario Mauri, Robert J. Omeljaniuk, Giuseppe Biagini, Antonio Torsello

**Affiliations:** 1 School of Medicine and Surgery, University of Milano-Bicocca, 20900 Monza, Italy; ramona.meanti@unimib.it (R.M.); m.criscione3@campus.unimib.it (M.L.C.); laura.rizzi@unimib.it (L.R.); elena.bresciani@unimib.it (E.B.); mario.mauri@unimib.it (M.M.); antonio.torsello@unimib.it (A.T.); 2 PhD Program in Translational and Molecular Medicine (DIMET), University of Milano-Bicocca, 20900 Monza, Italy; 3 Department of Biology, Lakehead University, Thunder Bay, ON P7B 5E1, Canada; romeljan@lakeheadu.ca; 4 Department of Biomedical, Metabolic, and Neural Sciences, University of Modena and Reggio Emilia, 41125 Modena, Italy

**Keywords:** neurosteroids, progesterone, epilepsy, neuroinflammation, microglial cells, GABA-A receptor, NLRP3 inflammasome, phagocytosis

## Abstract

**Background**: Progesterone (P4) is used as an antiseizure medication (ASM) to treat catamenial epilepsy, refractory to first-line drugs. P4 and other neurosteroids (NSs) are important regulators of multiple nervous system functions, including neuronal excitability and synaptic plasticity. In addition to their antiseizure properties, P4 and other NSs are also anti-inflammatory agents. Neuroinflammation is an important pathophysiological mechanism of epilepsy refractory to ASMs. Accordingly, we evaluated the ability of P4 to modulate neuroinflammation, using human microglia activated by lipopolysaccharide (LPS). **Methods**: Human microglia (HMC3) were stimulated for 3 h with LPS in the absence or presence of various concentrations of P4. Thereafter, levels of (i) toll-like receptor 4 (TLR4), (ii) the NLRP3 inflammasome, and (iii) pro-inflammatory cytokines were quantitated by real-time PCR and Western blot analyses. Phagocytic activity was also assessed using a phagocytosis assay employing fluorescent beads. **Results**: P4 treatment significantly reduced the microglial inflammatory state induced by LPS, which was mediated by upregulation of the TLR4- and NLRP3-axes. The protective effects of P4 were mediated by inhibition of Nuclear Factor kappa-light-chain-enhancer of activated B cells (NFκB) phosphorylation and reduced activation of Mitogen-Activated Protein Kinases (MAPK). The effects of P4 included a significant reduction in mRNA levels of the main pro-inflammatory cytokines and a reduction in phagocytic activity of HMC3. **Conclusions**: P4 is endowed with significant anti-inflammatory properties, which may be involved in the beneficial effects reported for drug-resistant catamenial epilepsy. Further research is required to clarify P4 post-receptor mechanisms of action and to explore the roles of other P4-derived NSs.

## 1. Introduction

Neuroinflammation is a pathophysiological process that occurs in response to noxious stimuli within the central nervous system (CNS) by promoting tissue repair in order to preserve neuronal integrity and brain function [1]. This process is primarily characterized by the activation of resident immune cells, microglia, and astrocytes, which play a central role in monitoring the CNS microenvironment and maintaining homeostasis under normal physiological conditions.

In the pro-inflammatory state induced by the toll-like receptor (TLR) ligand lipopolysaccharide (LPS), microglial cells initiate an inflammatory cascade by releasing many pro-inflammatory cytokines and mediators, including interleukin (IL)-1β, IL-6, IL-10, IL-18, tumor necrosis factor α (TNF-α), chemokines, and arginase 1 (Arg1), and activating the NLRP3 inflammasome complex [2]. The NLRP3 inflammasome constitutes a core component of the acute inflammatory response, which consists of a tightly regulated process that supports tissue repair and the restoration of homeostasis following insults [3]. While acute inflammation is generally beneficial and self-limiting, sustained microglial activation can lead to detrimental chronic neuroinflammation. This persistent state is marked by prolonged production of inflammatory mediators and is increasingly recognized as a major contributor to the development and progression of neurodegenerative diseases, including epilepsy [4,5,6,7].

Epilepsy is a severe neurological condition characterized by the brain’s predisposition to develop spontaneously recurrent seizures, leading to emotional and cognitive dysfunctions [8]. According to the International League Against Epilepsy (ILAE), epilepsy is defined as the occurrence of two unprovoked seizures more than 24 h apart, or a single unprovoked seizure when there is a high risk of recurrence [9]. Despite being one of the most common neurological disorders, affecting 50 million people worldwide, and the availability of various treatments, almost 30% of patients do not have any effective therapy [8]. In women, resistance to antiseizure medications (ASMs) could be related to the menstrual cycle, and, for this reason, this type of epilepsy is referred to as catamenial epilepsy. In some instances, catamenial epilepsy can be controlled by administering progesterone (pregn-4-ene-3,20-dione, P4), an endogenous neuroactive steroid hormone synthesized by the ovaries, placenta, adrenal glands and, locally, in the CNS by glia and neurons [10].

Thanks to its small size and lipid solubility, circulating P4 easily crosses the blood–brain barrier by free transmembrane diffusion throughout the CNS [11]. P4 and its metabolites are also known for their anxiolytic, antidepressant and neuroprotective effects. Moreover, several research studies have demonstrated the ability of P4 to modulate neuronal excitability by interacting with the progesterone receptors (PRs), progesterone receptor membrane component 1 (PGRMC1) and membrane progesterone receptor alpha (mPRα), and through the positive allosteric modulation of extrasynaptic gamma-aminobutyric acid (GABA)-A and N-methyl-D-aspartate (NMDA) receptors [12,13]. Interestingly, the decline in P4 during the late luteal phase is considered a major contributor to the perimenstrual increase in seizure susceptibility [14,15,16]. However, the specific underlying mechanisms of this phenomenon remain unclear.

The primary aim of this study was to explore the anti-inflammatory mechanisms of P4 on a human microglial cell line (HMC3 cells) stimulated with LPS, a well-established in vitro model of neuroinflammation. Our hypothesis is that P4 may reduce the human microglial inflammatory response and thereby suggest an additional mechanism explaining the ASM properties of this NS.

## 2. Results

### 2.1. Dose–Response Study with LPS for the Treatment of HMC3 Cells

HMC3 cells were incubated with various concentrations of LPS (30 ng/mL to 1 µg/mL) for 3 h in order to establish a reproducible inflammatory model.

**Figure 1:**
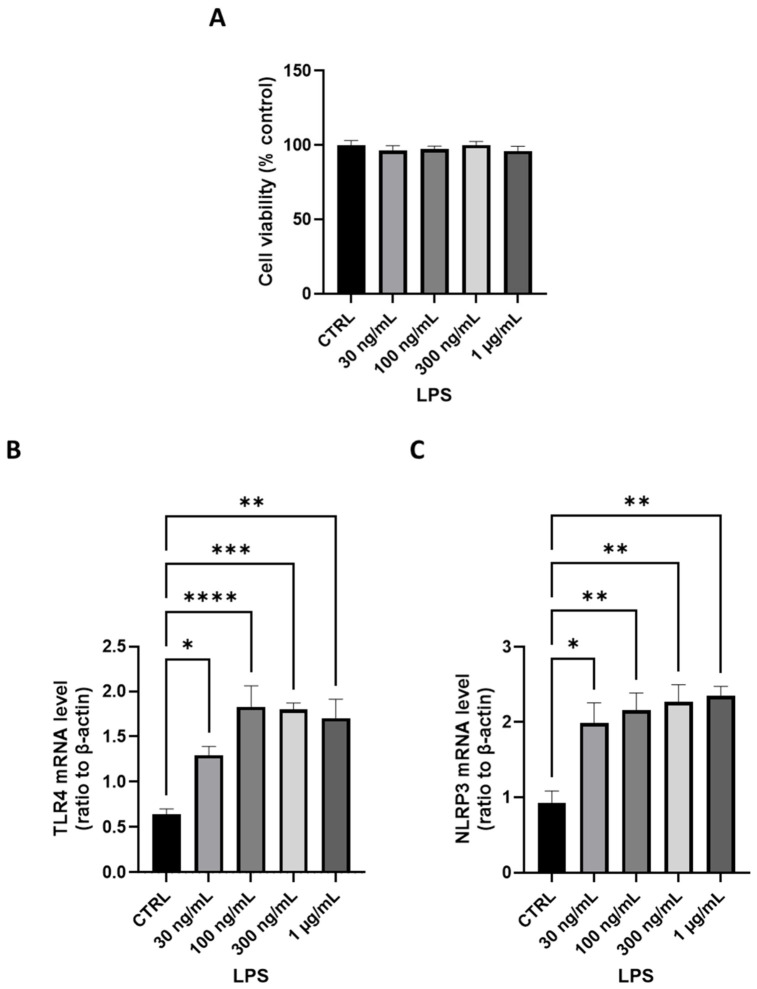

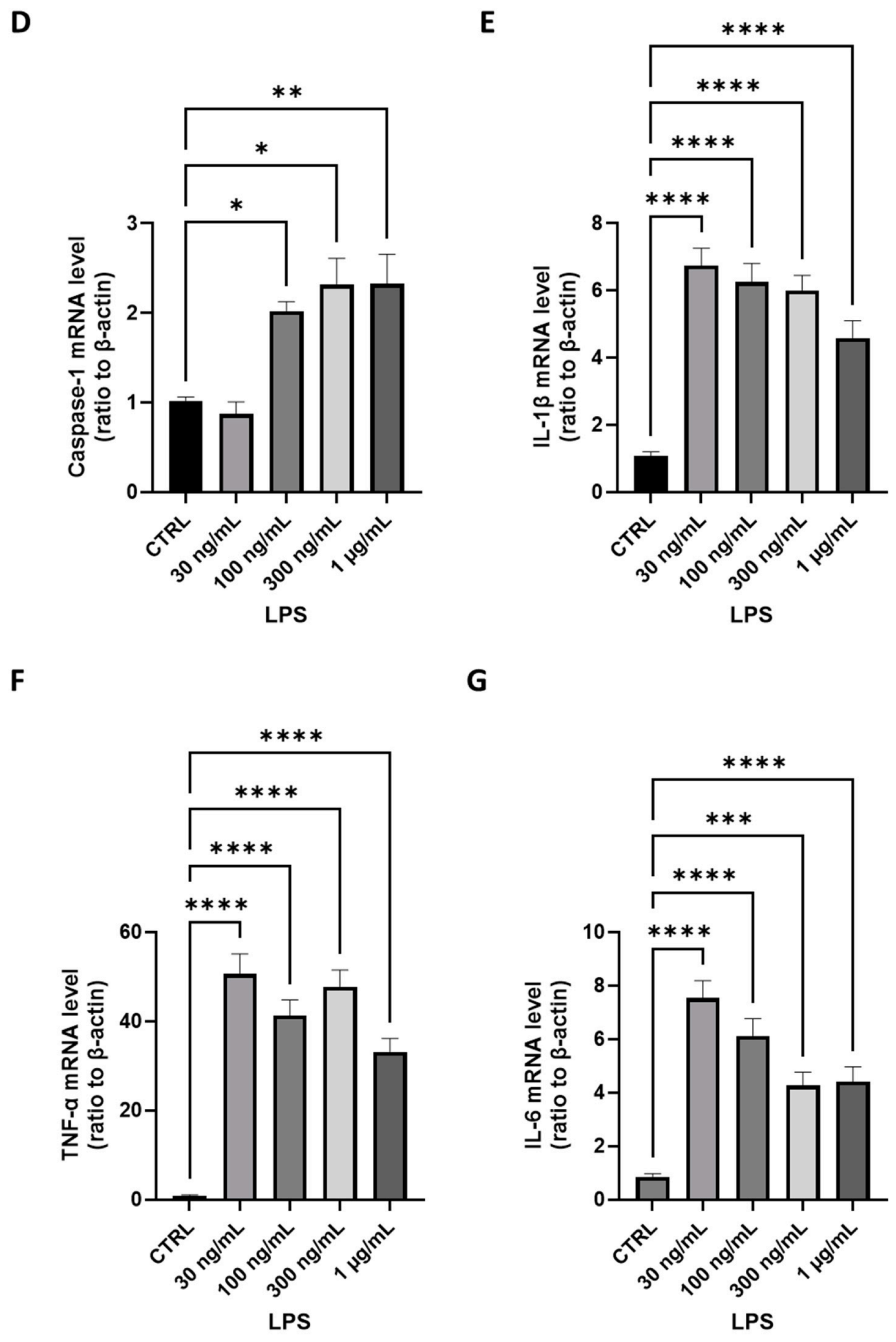
Effects of LPS on HMC3 proliferation and inflammatory mediators. HMC3 cells were plated at a density of 18,000 cells/well in 96-well plates or 110,000 cells/well in 24-well plates. The cells were treated for 3 h with increasing concentrations of LPS (30 ng/mL, 100 ng/mL, 300 ng/mL, 1 µg/mL). At the end of the treatment: (i) Cell proliferation was evaluated using a 3-(4,5-dimethylthiazol-2-yl)-2,5-diphenyltetrazolium bromide (MTT) assay (**A**). The results are expressed as mean (M) ± standard error of the mean (SEM) (*n* = 3 biological replicates, with a total of 15 technical replicates per condition; Tukey’s *t*-test). (ii) TLR4 (**B**), NLRP3 (**C**), Caspase-1 (**D**), IL-1β (**E**), TNF-α (**F**) and IL-6 (**G**) mRNA levels were evaluated by real-time PCR using β-actin as a housekeeping gene. The results are expressed as M ± SEM (*n* = 3 biological replicates, with a total of 18 technical replicates per condition). * *p* < 0.05, ** *p* < 0.01, *** *p* < 0.001, **** *p* < 0.0001, Tukey’s *t*-test.

LPS did not induce any cytotoxic effect on HMC3 cells and did not modify their proliferation rate at any of the concentrations tested (Figure 1A; Tukey’s *t*-test).

Real-time PCR revealed that incubation with LPS for 3 h induced a significant increase in mRNA levels for TLR4 (Figure 1B) and downstream effectors, suggesting a consequent inflammatory response in microglial cells (Figure 1C–G). LPS caused significant dose-dependent increases in NLRP3 (Figure 1C) and Caspase-1 mRNA levels (Figure 1D), wherein 100 ng/mL was the minimum effective LPS concentration capable of reproducibly inducing a maximal increase in the mRNA levels of these markers (TLR4: *p* < 0.0001; NLRP3: *p* < 0.01; Caspase-1: *p* < 0.05; Tukey’s *t*-test).

LPS also induced a significant (*p* < 0.0001; Tukey’s *t*-test) increase in the mRNA levels of IL-1β, TNF-α, and IL-6 (Figure 1E–G).

Based on these results, 100 ng/mL LPS was used for all the following experiments.

### 2.2. Dose–Response Study with P4 for the Treatment of HMC3 Cells

**Figure 2:**
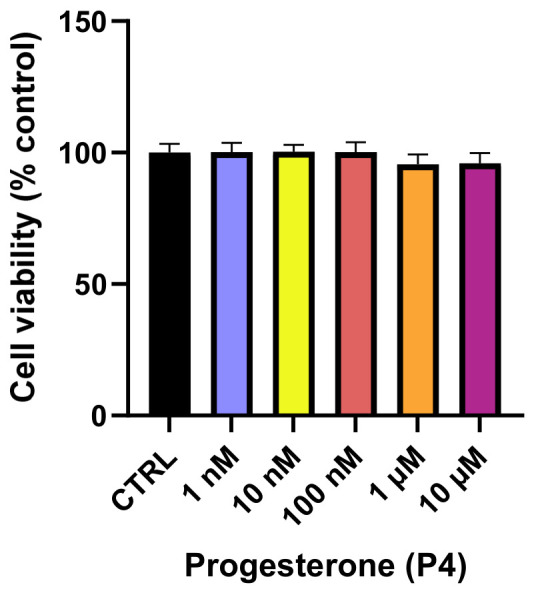
Effects of P4 treatment on HMC3 cell proliferation. HMC3 cells were plated at a density of 18,000 cells/well in 96-well plates. The cells were treated for 3 h with increasing concentrations of P4 (1 nM, 10 nM, 100 nM, 1 µM, 10 µM). At the end of the treatment, the cell number was evaluated compared to the control using an MTT assay. The results are expressed as the M ± SEM (*n* = 3 biological replicates, with a total of 15 technical replicates per condition; Tukey’s *t*-test).

HMC3 cells were incubated for 3 h with various concentrations of P4 (1 nM to 10 µM), followed by assessment of cell viability by MTT assay. The results show that treatment with P4 did not affect viability compared to the control group (Figure 2; Tukey’s *t*-test). In line with these results and with the literature, P4 at 10 µM was used in all the subsequent experiments.

### 2.3. Effects of P4 on TLR4 mRNA Levels and NFκB Phosphorylation in HMC3 Cells Following Treatment with LPS

Recent studies indicate that treatment with TLR4 antagonists may exert neuroprotective and anti-inflammatory effects in in vivo models of epilepsy, thereby suggesting that TLR4 represents a potential therapeutic target [17].

**Figure 3:**
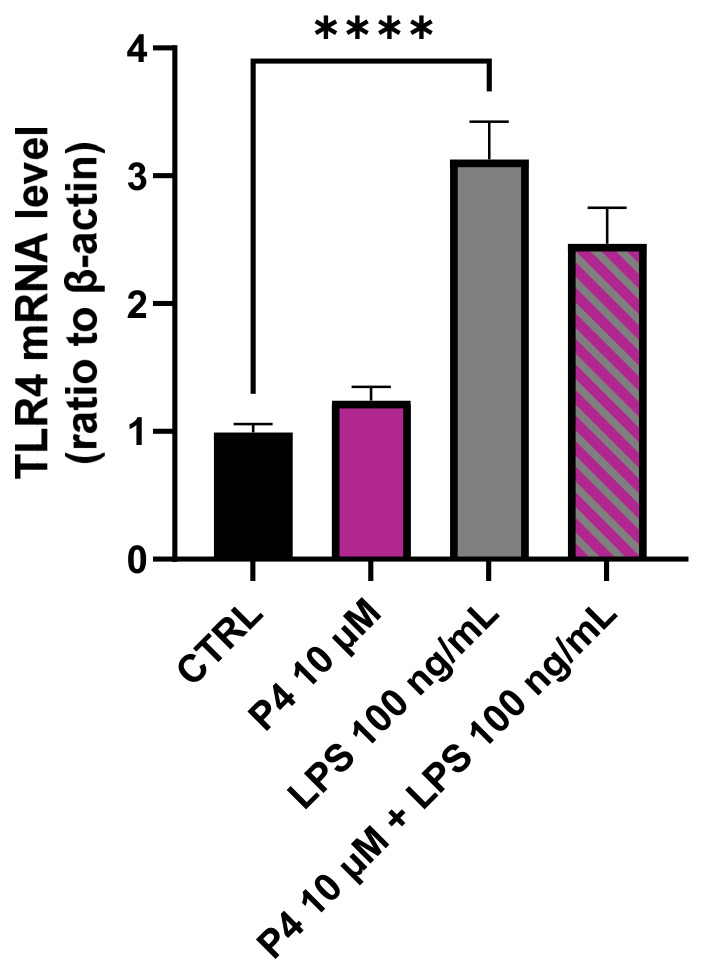
Effect of P4 on TLR4 mRNA levels in HMC3 cells. HMC3 were plated at a density of 110,000 cells/well in 24-well plates. The cells were treated for 3 h with LPS (100 ng/mL) and P4 (10 µM), either alone or in combination. At the end of the incubation, TLR4 mRNA expression was evaluated by real-time PCR, using β-actin as a housekeeping gene. The results are expressed as M ± SEM (*n* = 3 biological replicates, with a total of 18 technical replicates per condition). **** *p* < 0.0001, Tukey’s *t*-test.

As shown in Figure 3, LPS induced an increase in TLR4 mRNA levels (*p* < 0.0001; Tukey’s *t*-test), whereas P4 had a tendency to counteract LPS effects, although the differences did not reach statistical significance.

A class of transcription factors called Nuclear Factor kappa-light-chain-enhancer of activated B cells (NFκB) is involved in controlling inflammatory mediators and is located downstream of TLR signaling. In the inactive form, the NFκB subunits, p50 and p65, are complexed to IκBα in the cytoplasm. After the binding of LPS to TLR4, IκBα leaves the complex, enabling active NFκB (phosphorylated) to translocate into the nucleus and activate the transcription of pro-inflammatory cytokines and the expression of other genes linked to inflammation [18].

The activation of NFκB was determined by Western blot analysis, focusing in particular on the ratio of phosphorylated (active) to non-phosphorylated (inactive) states of NFκB.

**Figure 4:**
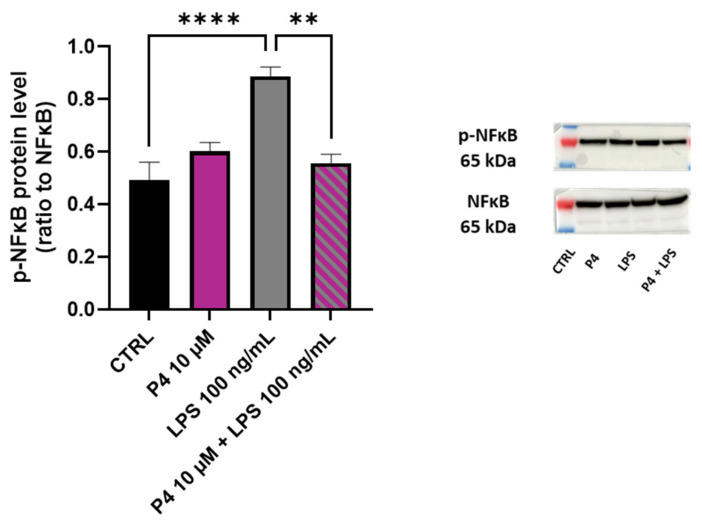
Effect of P4 on LPS-induced NFκB activation in HMC3 cells. HMC3 were plated at a density of 550,000 cells/well in 6-well plates and treated for 3 h with LPS (100 ng/mL) and P4 (10 µM), alone or in combination. At the end of the treatment, the cells were lysed, and the proteins were used for Western blot analysis of phosphorylated and non-phosphorylated forms of NFκB. β-actin was used for normalization of loading. The results are expressed as the M ± SEM (*n* = 3 biological replicates, with a total of 3 technical replicates per condition). ** *p* < 0.01, **** *p* < 0.0001, Tukey’s *t*-test.

The results show that treatment with LPS caused a significant increase in phosphorylated NFκB levels (Figure 4, *p* < 0.0001; Tukey’s *t*-test), while co-incubation with P4 significantly reduced the increase in the active form of NFκB caused by LPS (*p* < 0.01; Tukey’s *t*-test).

### 2.4. Effects of P4 on the Modulation of p38 and Extracellular Signal-Regulated Kinases (ERK 1/2) Phosphorylation in LPS-Treated HMC3 Cells

The Mitogen-Activated Protein Kinases (MAPK) pathway is widely involved in the regulation of cellular responses such as growth, survival, inflammation, and stress. In order to study the effects of P4 on the modulation of these signaling pathways, we quantified MAPK (ERK and p38) levels using Western blot analysis, focusing in particular on the ratio between the phosphorylated (active) and non-phosphorylated (inactive) forms of these proteins.

**Figure 5:**
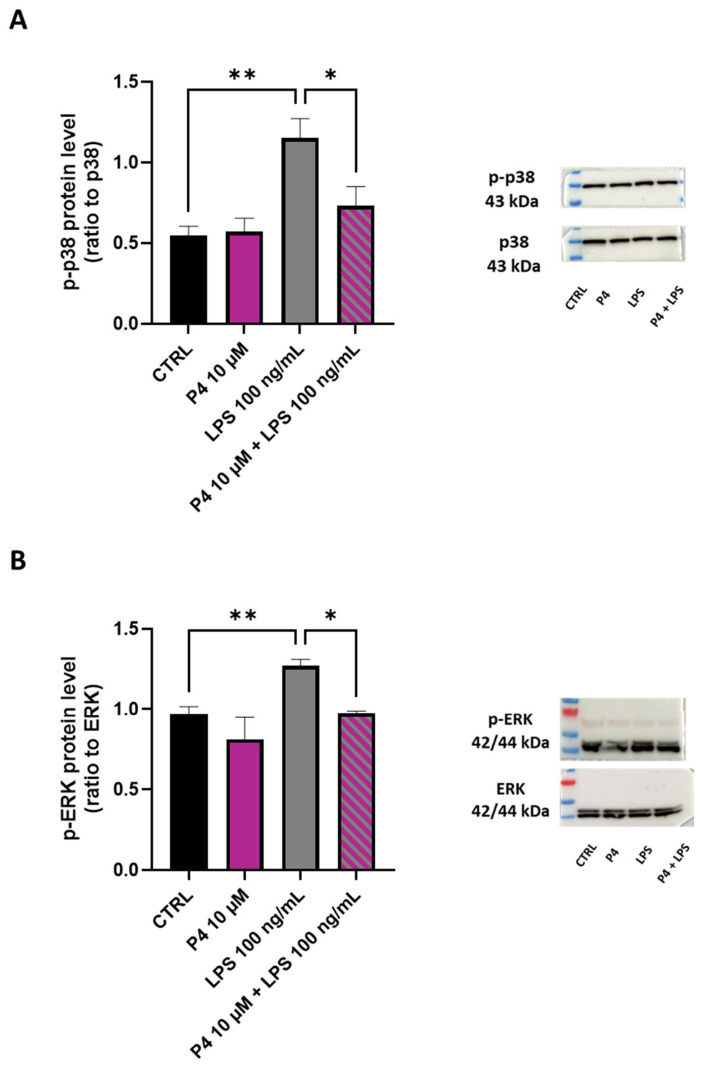
Variation in the phosphorylation levels of p38 and ERK1/2 proteins induced by LPS in HMC3 cells. HMC3 were plated at a density of 550,000 cells/well in 6-well plates and treated for 3 h with LPS (100 ng/mL) and P4 (10 µM), alone or in combination. At the end of the incubations, the cells were lysed, and the proteins were used for Western blot analysis of the phosphorylated and non-phosphorylated forms of p38 (**A**) and ERK1/2 (**B**). The results are expressed as the M ± SEM (*n* = 3 biological replicates, with a total of 3 technical replicates per condition). * *p* < 0.05 and ** *p* < 0.01, Tukey’s *t*-test.

Treatment of HMC3 cells with LPS increased the ratio of p-p38/p38 and of p-ERK/ERK (Figure 5A,B, *p* < 0.01; Tukey’s *t*-test). Co-incubation with P4 significantly reduced the degree of phosphorylation of both proteins (*p* < 0.05; Tukey’s *t*-test).

### 2.5. Effects of P4 on Modulation of the LPS-Induced NOD-, LRR- and Pyrin Domain-Containing Protein 3 (NLRP3) Inflammasome Pathway in HMC3 Cells

The inflammatory process plays a central role in the development of epilepsy; to illustrate, it has been reported that the activation of the NLRP3/Caspase-1 inflammasome complex and the consequent release of IL-1β and IL-18 cytokines exacerbates the epileptogenic state [19,20].

**Figure 6:**
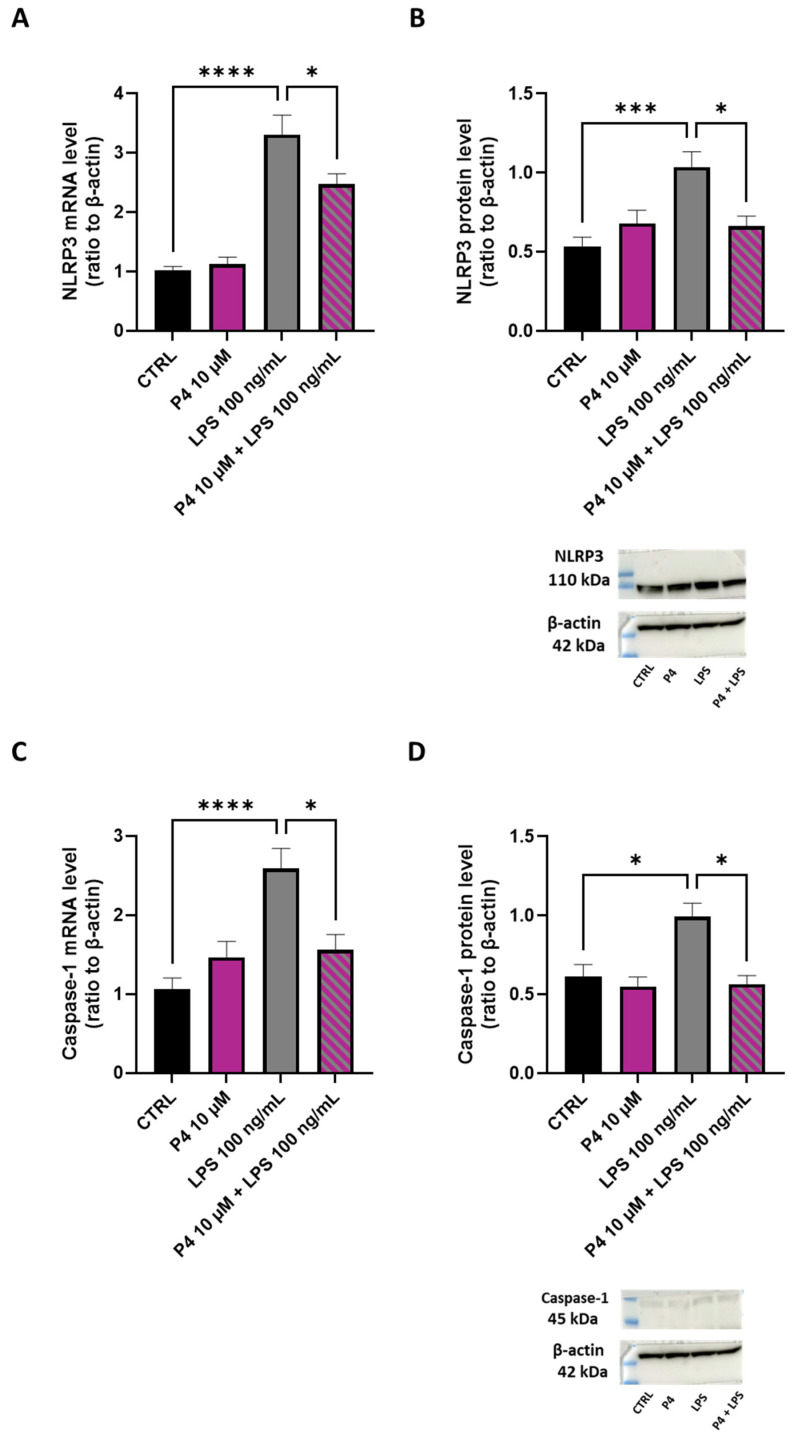

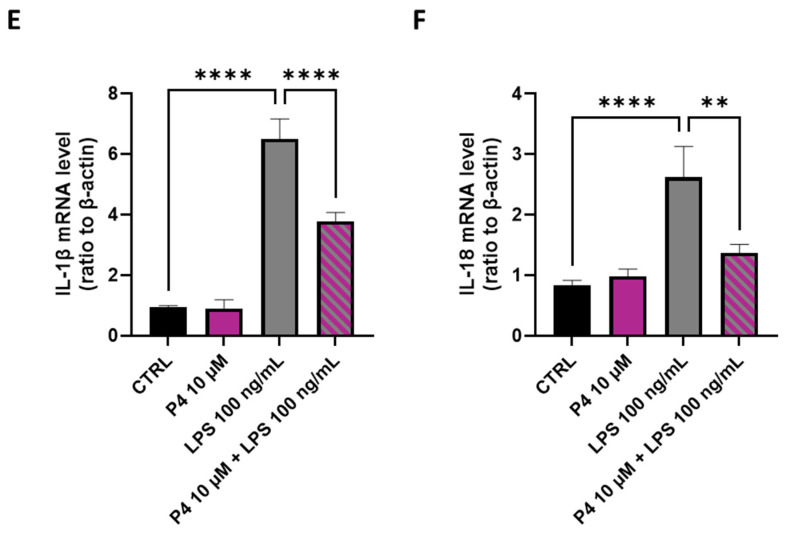
Effects of P4 on the modulation of the LPS-induced NLRP3 inflammasome pathway in HMC3 cells. HMC3 were plated at a density of 110,000 cells/well in 24-well plates. The cells were incubated for 3 h with LPS (100 ng/mL) and P4 (10 µM), either alone or in combination. At the end of the treatments, real-time PCR was used to evaluate the levels of NLRP3 (**A**), Caspase-1 (**C**), IL-1β (**E**), and IL-18 (**F**) mRNAs. β-actin was used as housekeeping gene. The results are expressed as M ± SEM (*n* = 3 biological replicates, with a total of 18 technical replicates per condition). * *p* < 0.05, ** *p* < 0.01 and **** *p* < 0.0001, Tukey’s *t*-test. For Western blot determinations, HMC3 cells were plated at a density of 550,000 cells/well in 6-well plates and treated for 3 h with LPS (100 ng/mL) and P4 (10 µM), alone or in combination. At the end of the treatment, the cells were lysed, and the proteins were used for Western blot analysis of NLRP3 (**B**) and Caspase-1 (**D**), and β-actin. The results are expressed as M ± SEM (*n* = 3 biological replicates, with a total of 3 technical replicates per condition). * *p* < 0.05, *** *p* < 0.001, Tukey’s *t*-test.

Treatment of HMC3 cells with P4 significantly reduced the mRNA and protein levels of both NLRP3 (Figure 6A,B, *p* < 0.05; Tukey’s *t*-test) and Caspase-1 (Figure 6C,D, *p* < 0.05; Tukey’s *t*-test) induced by LPS stimulation (NLRP3: *p* < 0.0001 for mRNA and *p* < 0.001 for protein; Caspase-1: *p* < 0.0001 for mRNA and *p* < 0.05 for protein; Tukey’s *t*-test).

Similarly, incubation with P4 for 3 h significantly attenuated the increase in mRNA levels of IL-1β and IL-18 (Figure 6E,F, *p* < 0.0001 and *p* < 0.01, respectively; Tukey’s *t*-test) induced by LPS (*p* < 0.0001; Tukey’s *t*-test).

These data suggest that the anti-inflammatory effects of P4 may be mediated by the inhibition of NLRP3/Caspase-1 cascades.

### 2.6. Effects of P4 on mRNA Levels of Pro-Inflammatory Cytokines in LPS-Treated HMC3 Cells

The M1 phenotype of microglia is associated with the production of pro-inflammatory cytokines such as IL-6, TNF-α, the inducible enzyme Nitric Oxide Synthase (iNOS) and Chemokine C-X-C motif ligand 1 (CXCL1).

**Figure 7:**
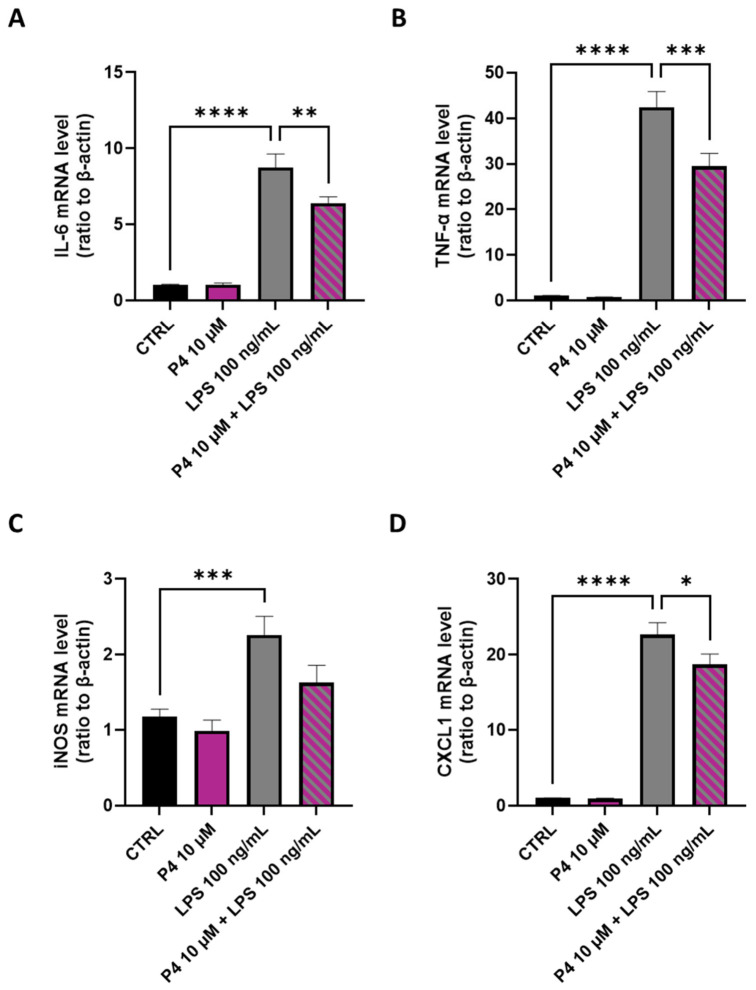
Effect of P4 on mRNA expression levels of IL-6, TNF-α, iNOS and CXCL1 induced by LPS treatment in HMC3 cells. HMC3 cells were plated at a density of 110,000 cells/well in 24-well plates. The cells were treated for 3 h with LPS (100 ng/mL) and P4 (10 µM), alone or in combination. At the end of the treatment, real-time PCR was used to evaluate the expression of TNF-α (**A**), IL-6 (**B**), iNOS (**C**) and CXCL1 (**D**) mRNA levels using β-actin as a housekeeping gene. The results are expressed as M ± SEM (*n* = 3 biological replicates, with a total of 18 technical replicates per condition). * *p* < 0.05, ** *p* < 0.01, *** *p* < 0.001 and **** *p* < 0.0001, Tukey’s *t*-test.

Incubation of HMC3 cells with LPS significantly increased the mRNA levels of IL-6, TNF-α, iNOS and CXCL1 (Figure 7A–D, *p* < 0.0001 for IL-6, TNF-α and CXCL1; *p* < 0.001 for iNOS; Tukey’s *t*-test).

Treatment with P4 also significantly antagonized the expression of IL-6 (*p* < 0.01; Tukey’s *t*-test), TNF-α (*p* < 0.001; Tukey’s *t*-test) and CXCL1 (*p* < 0.05; Tukey’s *t*-test) compared to the group treated with LPS alone and appeared to reduce the mRNA levels of iNOS (increased by treatment with LPS). Incubation with P4 alone did not modify the cytokine mRNA levels compared to the control group, which suggests that P4 inhibitory effects are specific to the LPS-induced inflammatory conditions.

### 2.7. Effects of P4 on Phagocytic Activity in LPS-Treated HMC3 Cells

**Figure 8:**
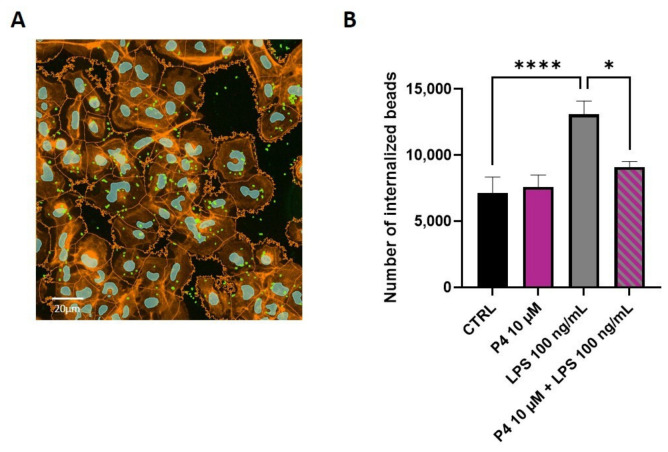
Effect of P4 on HMC3 phagocytic activity. (**A**) HMC3 cells were seeded on poly-D-lysine pre-treated coverslips at a density of 80,000 cells/well in 24-well plates and incubated for 3 h with or without 10 µM P4 and 100 ng/mL LPS. At the end of the treatment, the cells were incubated with fluorescent beads for 2 h, then fixed and stained for phalloidin and DAPI. Representative image of a processed field with ARIVIS Pro software (v4.4) showing, in orange, the segmented cell body; in cyan, the nuclei; and in green, the beads. (**B**) Graphical representation of the number of internalized beads obtained by the calculation of the total number of beads phagocytized. Data are expressed as M ± SEM (*n* = 3 biological replicates, with a total of 9 coverslips per condition; 30 representative fields were captured for each coverslip). * *p* < 0.05 and **** *p* < 0.0001, Tukey’s *t*-test.

HMC3 cells were incubated with P4 (10 μM) in the absence or presence of LPS (100 ng/mL) in order to investigate the effects of P4 on LPS-induced microglial activation. Figure 8A shows a representative image of the AI-driven method of analysis. LPS stimulated a significant increase (*p* < 0.0001; Tukey’s *t*-test) in the number of phagocytosed beads (Figure 8B) in comparison to controls, and this effect was reduced by co-incubation with P4. These results suggest that P4 antagonizes LPS-induced phagocytic activity (*p* < 0.05; Tukey’s *t*-test) and therefore modulates the microglial phenotype from a pro-inflammatory M1 to an anti-inflammatory M2, as also suggested by the real-time PCR data reported above.

## 3. Discussion

This study demonstrates that P4 exerts relevant anti-inflammatory effects by targeting the TLR4/NLRP3 axis and its downstream NFκB and MAPK signaling pathways, as well as modulating microglial functional phenotype.

It has been proposed that P4 effects are mediated by non-classical progesterone signaling pathways. In particular, PGRMC1 and mPRs are known to mediate rapid, non-genomic progesterone signaling and could interact functionally. Importantly, the loss of PGRMC1 has been shown to exacerbate LPS-induced NFκB activation and cytokine production, suggesting a role in restraining neuroinflammation [21,22]. Furthermore, a study conducted using LPS-stimulated murine BV2 microglial cells demonstrated that P4 exerts pleiotropic anti-inflammatory effects mediated by PR receptors and the consequent modulation of NFκB and MAPK signaling pathways, leading to down-regulation of TNF-α, iNOS and COX-2 [23]. Our experimental data (Supplementary Figure S1 in Supplementary Materials) show that HMC3 cells express both the PGRMC1 and the mPRα (also known as PAQR7), but not the classical progesterone receptor (PGR).

At the same time, both progesterone and its metabolites may modulate the activation of GABA-A receptors, which are expressed in the HMC3 cell line, and that could contribute, to some extent, to the anti-inflammatory effects of progesterone observed in this study [24].

Chronic neuroinflammation is a hallmark of several neurological disorders, including drug-resistant epilepsy, and is characterized by sustained activation of microglia and excessive production of pro-inflammatory mediators. Increasing evidence indicates that inflammatory signaling, particularly via innate immune receptors and inflammasomes, critically contributes to seizure susceptibility, neuronal injury, and disease progression [8,25,26,27]. Therefore, modulation of microglial inflammatory pathways represents a promising therapeutic strategy, with anti-inflammatory and tissue-protective outcomes.

The mechanism of action of progesterone is not completely understood and could be complex, like in monocytes. In circulating monocytes, progesterone primarily acts as a context-dependent modulator of acute inflammatory responses. Upon toll-like receptor stimulation, progesterone suppresses pro-inflammatory cytokine production (TNF-α, IL-6, IL-1β) through inhibition of NFκB and MAPK signaling, effectively limiting systemic innate immune activation. Additional evidence indicates that P4 attenuates TLR4-mediated MyD88-dependent signaling by downregulating surface TLR4 expression on monocytes, reducing cellular responsiveness to LPS stimulation [28]. However, this anti-inflammatory effect is not always true since in other monocytic models, progesterone increased cytokine release, indicating that monocytes retain a flexible, stimulus-dependent response to progesterone. Thus, in monocytes, progesterone behaves as a tunable regulator, capable of either dampening or stimulating inflammation, depending on the context [28,29,30].

A key mechanistic study demonstrated that P4 pre-treatment significantly suppresses TLR4-triggered production of IL-6 and nitric oxide in macrophages, acting by downregulating TLR4 surface expression, inhibiting NFκB phosphorylation, and upregulating SOCS1, a negative feedback regulator of cytokine signaling [31]. Consistent with this, P4, acting via its receptors, more broadly suppresses NFκB activation downstream of TLR4, thereby reducing the transcription of pro-inflammatory mediators such as IL-6, TNF-α, and IL-1β [32,33].

Our findings are consistent with previous reports in which LPS activated microglia through TLR4, consequently triggered NFκB and MAPK pathways and promoted inflammasome activation [34]; specifically, our data confirm that LPS markedly increases TLR4 mRNA expression in HMC3 cells. This LPS effect was accompanied by enhanced activation of the NLRP3 inflammasome complex, as evidenced by increased mRNA and protein levels of NLRP3 and caspase-1 and by increased mRNA levels of IL-1β and IL-18. The NLRP3 inflammasome is one of the most extensively characterized inflammasomes in the CNS. The NLRP3 inflammasome plays a central role in the maturation of IL-1β and IL-18 and consequent amplification of inflammatory cascades. These inflammatory cascades contribute to neuronal pathologies associated with (i) epilepsy, (ii) multiple sclerosis, (iii) Parkinson’s disease, (iv) amyotrophic lateral sclerosis, and (v) Alzheimer’s disease [35]. In addition, inflammasome activation promotes pyroptosis, which further exacerbates neuroinflammation.

Notably, P4 could cause a tendency toward a reduction in LPS-induced TLR4 mRNA expression. Although this is not statistically significant, it implies the possibility of an upstream regulatory effect on innate immune sensing. This finding is in line with the literature, where it is demonstrated that P4 plays a minor role in the direct inactivation of TLR4 but inhibits its inflammatory pathway (NFkB, MAPK and pro-inflammatory cytokines) [28,36,37]. In fact, we measured a marked downregulation of NLRP3 and caspase-1 at both mRNA and protein levels, and of IL-1β and IL-18 mRNA levels, indicating that P4 interferes with inflammasome priming and activation. These findings are consistent with accumulating evidence that P4 exerts anti-inflammatory and neuroprotective effects in various models of CNS injury and inflammation by modulating microglial activation and inflammasome signaling [38,39].

LPS treatment induced phosphorylation of NFκB p65 and MAPKs p38 and ERK, which is consistent with activation of canonical pro-inflammatory pathways downstream of TLR4. P4 significantly attenuated the phosphorylation of NFκB, p38, and ERK, without affecting the total protein levels. NFκB and MAPKs are well-established mediators of LPS-induced transcription of TNF-α, IL-6, iNOS, and chemokines such as CXCL1 [23]. Accordingly, we observed that LPS robustly upregulated mRNA expression of TNF-α, IL-6, iNOS, and CXCL1, whereas P4 significantly reduced their expression, thereby confirming its ability to dampen the pro-inflammatory transcriptional program in activated human microglia.

In addition to cytokine production, microglial activation is associated with functional changes, including enhanced phagocytic activity. In our model, LPS increased the phagocytosis of fluorescent beads by HMC3 cells, thereby illustrating the phenotypic plasticity of this microglial cell. Notably, P4 reduced LPS-induced phagocytic activity, which suggests that P4 not only modulates inflammatory signaling but also reshapes microglial functional state [40,41].

Collectively, our findings identify P4 as a potent modulator of LPS-induced neuroinflammation in human microglia. By downregulating TLR4 expression, inhibiting NFκB and MAPK phosphorylation, suppressing NLRP3 inflammasome activation, and reducing the expression of pro-inflammatory mediators and phagocytic activity, P4 effectively counteracts key molecular and functional hallmarks of microglial activation. These results may provide some indications about the anti-inflammatory action of P4 on microglia.

## 4. Materials and Methods

### 4.1. Cell Culture

HMC3 cells (ATCC^®^CRL-330, Manassas, VA, USA) are a cell line derived from SV40-dependent immortalization of human embryonic microglial cells. The HMC3 cells were grown in Dulbecco’s Modified Eagle Medium high glucose (DMEM high glucose, Sigma-Aldrich, St. Louis, MO, USA) supplemented with 15% heat-inactivated FBS (Euroclone, Pero, Milan, Italy), 100 IU/mL penicillin (Euroclone), 100 µg/mL streptomycin (Euroclone) and Mycozap (Euroclone), under standard cell culture conditions (37 °C, 5% CO_2_). After reaching confluence, the HMC3 cells were washed with PBS, detached with trypsin-EDTA solution (Euroclone), and seeded for experiments.

In each experiment, the HMC3 cells were incubated for 3 h with lipopolysaccharide (LPS, 100 ng/mL, L4005, Sigma-Aldrich) alone or in combination with progesterone (P4, 10 µM, P8783, Sigma-Aldrich).

### 4.2. Cell Viability

To assess the cytotoxicity of the treatments and the resulting cell viability, the HMC3 cells were seeded into 96-well culture plates (18 × 10^3^ cells/well), and the following day, they were incubated with various concentrations (30 ng/mL, 100 ng/mL, 300 ng/mL, 1 µg/mL) of LPS or P4 (1 nM, 10 nM, 100 nM, 1 µM, 10 µM) for 3 h. At the end of the treatment, 10 µL of MTT (5 mg/mL, M5655, Sigma-Aldrich) was added to each well, and the plate was incubated at 37 °C for 3 h. The formazan crystals were dissolved by removing the culture medium and adding 200 µL of acidified isopropanol. Absorbance was read at 570 nm using the multilabel spectrophotometer Spectramax ID5 (Molecular Devices, San Jose, CA, USA). After setting the cell viability of the control group at 100%, the values for the experimental groups were calculated as follows:

$$\%\;\mathrm{of}\;\mathrm{viable}\;\mathrm{cells}=\frac{absorbance\;of\;experimental\;group}{absorbance\;of\;relative\;control}\times 100.$$

### 4.3. Real-Time PCR Analysis

In order to assess the mRNA levels of the markers of interest, HMC3 cells were plated in 24-well culture plates at a density of 1.1 × 10^5^ cells/well and treated for 3 h with LPS and P4 according to previously described protocols. At the end of the treatment, the culture medium was removed. The total RNA was extracted following the instructions for EZ2 RNA/miRNA Tissue/Cell Kit (QIAGEN, Hilden, Germany) and quantified with a Nanodrop ND-1000 spectrophotometer (Thermo Fisher Scientific, Waltham, MA, USA). Following enzymatic digestion and reverse transcription using an iScript cDNA Synthesis Kit (Bio-Rad, Hercules, CA, USA), the cDNA amplification (21 ng) was performed in a total volume of 10 μL of iTaq Universal Probes Supermix (Bio-Rad) using the standard program of Fast real-time PCR System 7900HT (Applied Biosystems, Waltham, MA, USA). β-actin was used as internal control and to normalize the relative mRNA concentrations of the target genes, which were calculated using the 2^−ΔΔCt^ method.

### 4.4. Western Blot Analysis

For the quantification of specific proteins, HMC3 cells were plated in 6-well culture plates at a density of 5 × 10^5^ cells/well, incubated at 37 °C for 24 h and then treated as previously described. Cell lysates were prepared in RIPA buffer (Cell Signaling Technology, Danvers, MA, USA) with protease inhibitors, and the total protein concentrations were quantified using a Pierce BCA Protein Assay Kit (Thermo Fisher Scientific). A total of 50 µg of total proteins were loaded and run on precast 4–12% gradient gels (Invitrogen, Waltham, MA, USA), separated by electrophoresis, blotted on polyvinylidene difluoride (PVDF) membranes (Thermo Fisher Scientific), and blocked in 5% non-fat milk or BSA [42,43]. The incubation of primary antibodies was run overnight at 4 °C: p38 MAPK p40 XP Rabbit mAB, 1:1000; Phospho-p38 MAPK p43 XP Rabbit mAB, 1:1000; MAPK (ERK 1/2) XP Rabbit mAB p44/42, 1:1000; Phospho-MAPK (ERK 1/2) XP Rabbit mAB, 1:1000; NFκB XP Rabbit mAB, 1:1000; Phospho-NFκB XP Rabbit mAB p65, 1:1000; NLRP3 XP Rabbit mAB, 1:1000; β-actin XP Rabbit mAB, 1:2500 (all Cell Signaling Technology); Casp1 XP Rabbit pAB, 1:1000 (OriGene, Rockville, MD, USA). The peroxidase-conjugated goat anti-rabbit IgG, 1:5000 (Thermo Fisher Scientific) secondary antibody was incubated for 1 h at room temperature. Chemiluminescence signals were detected using LiteAblot TURBO (Euroclone) and detected with Amersham ImageQuant 800 (GE Healthcare, Chalfont St Giles, Buckinghamshire, UK). Image J software (1.48v) was used to quantify the protein bands.

### 4.5. Phagocytosis Assay

HMC3 cells (8 × 10^4^ cells/well) were seeded onto poly-D-lysine-coated coverslips (P0899, Sigma-Aldrich) and incubated for 24 h at 37 °C. The cells were then treated with 100 ng/mL LPS for 3 h, either with or without P4 (10 µM). Approximately 5 μL/mL of fluorescence-labeled latex beads (L4655, Sigma-Aldrich) were added for 2 h at 37 °C. The cells were then washed three times with PBS to remove the excess beads and fixed with 4% paraformaldehyde (Titolchimica, Rome, Italy). To label the cell membranes and nuclei, the HMC3 cells were incubated with 1 U/mL Alexa Fluor 594 phalloidin (A12381, Thermo Fisher Scientific) and with 1 µg/mL DAPI (D9542, Sigma-Aldrich), respectively. Cellular images were acquired with an inverted microscope (Axio Observer, ZEISS, Jena, Germany); 30 representative fields were captured at 40x magnification with random distribution along the coverslip with ZEN software (3.5 blue edition) (ZEISS, Oberkochen, Germany), keeping all the imaging parameters constant.

Segmentation of the cell bodies, nuclei, and bead signals, followed by quantitative analysis, was conducted using ZEISS Arivis Pro (v4.4, ZEISS, Germany). A custom analysis pipeline was implemented after a fast preprocessing comprising background subtraction and 3D Gaussian filter for noise attenuation, and we performed a CellPose-based segmentation for the nuclei, the ‘Blob Finder’ algorithm for precise bead identification, and a region-growing algorithm with watershed and threshold adjustment for cell body reconstruction.

The complete pipeline is available in the Supplementary Materials.

The number of beads internalized by the HMC3 cells following treatments was thus quantified and normalized, taking into account the total number of beads present in the photomicrograph (n° internalized beads = n° total beads − n° beads in cell shape).

### 4.6. Statistical Analysis

GraphPad Prism 10 (GraphPad Software, San Diego, CA, USA) was used for statistical analysis. The data are expressed as the mean (M) ± standard error of the mean (SEM). At least three independent replications of the experiments were conducted. Statistical analysis was performed by one-way ANOVA for multiple comparisons, followed by Tukey’s *t*-test. A *p*-value lower than 0.05 was considered significant.

## 5. Conclusions

Although the present study was performed in an in vitro model of human microglia, we speculate that our findings provide mechanistic insight into the anti-inflammatory effect of P4 also observed in vivo in epileptic rat models and in the clinical approach to the treatment of catamenial epilepsy [44,45,46,47,48]. This study has partially addressed knowledge gaps by some aspects of the molecular mechanisms involved. In fact, P4 is able to exert neuroprotective and anti-inflammatory action in HMC3 microglial cells, reducing neuroinflammation induced by LPS treatment.

New investigations are needed to obtain a complete picture of the molecular mechanisms underlying the neuroprotective and anti-inflammatory actions of P4 and to test other NSs derived from P4 metabolism.

## Data Availability

The original contributions presented in this study are included in the article/Supplementary Material. Further inquiries can be directed to the corresponding authors.

## Supplementary Materials

The following supporting information can be downloaded at: https://www.mdpi.com/article/10.3390/ph19060920/s1, Supplementary Figure S1: mRNA expression levels of PGR, PGRMC1 and PAQR7 in HMC3 cells. HMC3 cells were plated at a density of 110,000 cells/well in 24-well plates and cultured for 24 hours. After harvesting, total RNA was obtained as described in the Section 4. Real-time PCR was used to evaluate the expression of three different P4 receptors: PGR, PGRMC1 and PAQR7. The relative mRNA levels were calculated using β-actin as a housekeeping gene. Results are the mean ± SEM (*n* = 3 biological replicates, with a total of 12 technical replicates per condition; Tukey’s *t*-test). Supplementary Material S1: Complete custome analysis pipeline created and used for the quantification of phagocytes beads.

## Abbreviations

The following abbreviations are used in this manuscript:

Arg1	Arginase 1
ASM	Antiseizure medication
BCA	Bicinchoninic acid
BSA	Bovine Serum Albumin
Casp1	Caspase-1
CNS	Central Nervous System
DAPI	4′,6-diamidino-2-phenylindole
DMEM	Dulbecco’s Modified Eagle Medium
DRE	Drug-resistant epilepsy
ERK	Extracellular signal-regulated kinase
FBS	Fetal Bovine Serum
GABA	γ-aminobutyric acid
HMC3	Human microglial cells
IL-1β	Interleukin-1 beta
IL-6	Interleukin-6
IL-10	Interleukin-10
IL-18	Interleukin-18
ILAE	International League Against Epilepsy
iNOS	Inducible Nitric Oxide Synthase
LPS	Lipopolysaccharide
MAPK	Mitogen-activated protein kinases
mPRs	Membrane progesterone receptors
mPRα	Membrane progesterone receptor alpha (alias PAQR7)
MTT	3-(4,5-Dimethylthiazol-2-yl)-2,5-diphenyl tetrazolium bromide
NFκB	Nuclear factor kappa-light-chain-enhancer of activated B cells
NLRP3	Nucleotide-Binding Domain, Leucine-Rich Repeat Domain, and Pyrin Domain-Containing Protein 3
NMDA	N-methyl-D-aspartate
NSs	Neurosteroids
P4	Progesterone (pregn-4-ene-3,20-dione)
p38	p38 mitogen-activated protein kinase
PAQR7	progestin and adipoQ receptor family member 7 (alias mPRα)
PBS	Phosphate-buffered saline
PBS-T	Phosphate-buffered saline with Tween-20
PGR	Progesterone receptor
PGRMC1	Progesterone receptor membrane component 1
p-p38	Phosphorylated p38
PRs	Progesterone receptors
PVDF	Polyvinylidene difluoride
RIPA	Radioimmunoprecipitation assay buffer
TLR	Toll-like receptor
TNF-α	Tumor necrosis factor α
