# Supplementary material for: Anti-Inflammatory Effects of Progesterone on Human Microglia via TLR4/NLRP3 Pathway Modulation: Relevance to Drug-Resistant Epilepsy
Source: Pharmaceuticals (Basel). 2026 Jun 11;19(6):920. doi: 10.3390/ph19060920 (PMC13306037; doi:10.3390/ph19060920)
Supplement: Supplementary file 1 [file pharmaceuticals-19-00920-s001.zip › pharmaceuticals-4279712-supplementary.pdf]

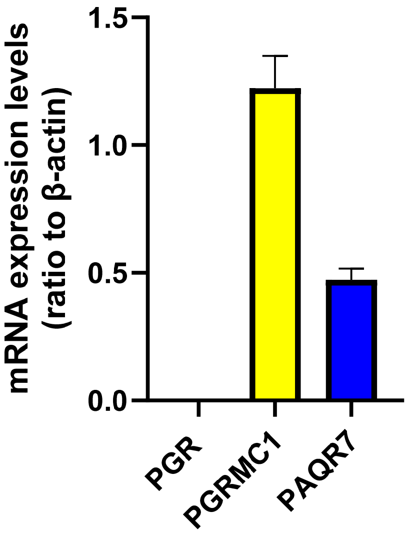

Supplementary Figure S1. mRNA expression levels of PGR, PGRMC1 and PAQR7 in HMC3 cells. HMC3 cells were plated at a density of 110,000 cells/well in 24-well plates and cultured for 24 hours. After harvesting, total RNA was obtained as described in the Methods section. Real-time PCR was used to evaluate the expression of three different P4 receptors: PGR, PGRMC1 and PAQR7. The relative mRNA levels were calculated using β-actin as a housekeeping gene. Results are the mean ± SEM (n=3 biological replicates, with a total of 12 technical replicates per condition; Tukey's t-test).

Supplementary Material S1. Complete custome analysis pipeline created and used for the quantification of phagocytes beads.

<!--Created by arivis Pro 4.4.0 tags/4.4-release^0@de835d9cac6666dddbfbb4871fddf807d9922d0f-->

<pipeline created="2025-03-25T10:24:02.0946699Z" modified="2025-11-17T11:41:00.0086636+01:00" version="4.4" read_version="4.2">

<operations>

<operation type-id="arivis.analysis.selection" id="arivis.analysis.selection1">

<outputs>[{"Channels":[{"v":1,"id":"arivis.analysis.selection1.0.0","p":null,"n":"DAPI","a":true},{"v":1,"id":"
arivis.analysis.selection1.0.1","p":null,"n":"FITC","a":true},{"v":1,"id":"arivis.analysis.selection1.0.2","p":null
,"n":"DsRed","a":true}]},{"Channels":[]},{"ObjectTags":[]}]</outputs>

<settings>{"FilePath":"E:\\ARIVIS\\Meanti\\251105\\ctrl_3.sis","ImageSetId":"0","PixelSize":{"X":0.517500
0000000001,"Y":0.5175000000000001,"Z":1.0},"PixelType":"UInt8","ChannelCount":3,"Bounds":{"X1":0,"X
2":639,"Y1":0,"Y2":671,"Z1":0,"Z2":0,"T1":0,"T2":29},"FullBounds":{"X1":0,"X2":639,"Y1":0,"Y2":671,"Z1":0,
"Z2":0,"T1":0,"T2":29},"Channels":"1;2;3","SelectionHint":"CurrentImageSet","Scaling":1.0,"ScalingIncludeZ
":true,"CropInputData":false,"LegacyMode":false}</settings>

</operation>

<operation type-id="arivis.analysis.segmentation.CellposeSegmentationOperation" id="arivis.analysis.segmentation.CellposeSegmentationOperation1" name="Segmentatio_nucleiu">

<inputs>[{"Channels":[{"v":1,"id":"arivis.analysis.selection1.0.0"}]},{"Channels":[]}]</inputs>

<outputs>[{"ObjectTags":[{"v":1,"id":"arivis.analysis.segmentation.CellposeSegmentationOperation1.0.0","
p":null,"n":"Nuclei","a":true,"cm":2,"c":"#00FFFF"}]}]</outputs>

<settings>{"Model":"CP","ExternalModelFilePath":null,"DiameterActive":false,"UseOptimizedBorderOverla
p":false,"Diameter":8.79,"LowerQuantile":0.01,"UpperQuantile":0.99,"FlowThreshold":0.4,"ProbabilityThre
shold":0.0,"MinSizeFilterActive":false,"MinSize":{"Value":5.5485E+18,"Type":1},"PerformPlaneWise":true,"
Connectivity":"FullXY","AllowHoles":false,"ObjectNamePattern":"$t #$I
($o)","SmoothNetworkOutput":true,"SmoothNetworkOutputSigma":2.4,"UseFlowThreshold":true}</settin
gs>

</operation>

<operation type-id="arivis.analysis.segmentation.BlobFinder" id="arivis.analysis.segmentation.BlobFinder1" name="Blob Finder">

<inputs>[{"Channels":[{"v":1,"id":"arivis.analysis.selection1.0.1"}]}]</inputs>

<outputs>[{"ObjectTags":[{"v":1,"id":"arivis.analysis.segmentation.BlobFinder1.0.0","p":null,"n":"particles",
"a":true,"cm":2,"c":"#00D800"}]}]</outputs>

<settings>{"Diameter":1.1499999999999997,"Level":0.04103947054000219,"Threshold":0.1641378104686

737,"Normalization":"PerTimePoint","Minimum":6.0,"Maximum":223.0,"PerformPlaneWise":false,"Connectivity":"FullXY","AllowHoles":false,"ObjectNamePattern":"$t #$I
($o)","UseExponentialNormalization":true}</settings>

</operation>

<operation type-id="arivis.analysis.volume.FeatureFilter" id="arivis.analysis.volume.FeatureFilter1" name="Object Feature Filter">

<inputs>[{"ObjectTags":[{"v":1,"id":"arivis.analysis.segmentation.BlobFinder1.0.0"}]}]</inputs>

<outputs>[{"ObjectTags":[{"v":1,"id":"arivis.analysis.volume.FeatureFilter1.0.0","p":null,"n":"Particles Filter","a":true,"cm":2,"c":"#6BD8B3"},{"v":1,"id":"arivis.analysis.volume.FeatureFilter1.0.1","p":null,"n":"not Object Feature Filter","a":false,"cm":0,"c":"Cyan"}]}]</outputs>

<settings>{"FilterParts":[{"Mode":"Type","Feature1":{"FeatureId":"arivis.analysis.features.type","ValueIndex":0},"Feature2":null,"Comparison":"GreatherThan","CompareValue1":0.0,"CompareValue2":1.0,"CompareString":""},{"Mode":"SingleFeature","Feature1":{"FeatureId":"arivis.analysis.features.projected-properties","ValueIndex":0},"Feature2":null,"Comparison":"GreatherThan","CompareValue1":1.6999999999999998E-12,"CompareValue2":1.0,"CompareString":""}]}</settings>

</operation>

<operation type-id="arivis.analysis.segmentation.RegionGrowing" id="arivis.analysis.segmentation.RegionGrowing1" name="Region Growing">

<inputs>[{"ObjectTags":[{"v":1,"id":"arivis.analysis.segmentation.CellposeSegmentationOperation1.0.0"}]},{"Channels":[{"v":1,"id":"arivis.analysis.selection1.0.2"}]}]</inputs>

<outputs>[{"ObjectTags":[{"v":1,"id":"arivis.analysis.segmentation.RegionGrowing1.0.0","p":null,"n":"Cell_shape","a":true,"cm":2,"c":"#FF8000"}]}]</outputs>

<settings>{"Connectivity":"FullXY","AllowHoles":false,"PerformPlaneWise":false,"ObjectNamePattern":"$t
#$I
($o)","RGMethod":"Watershed","MaskSeeds":false,"MaximumDistance":26.910000000000004,"Threshold":19.0,"LowContrastBorders":true}</settings>

</operation>

<operation type-id="arivis.analysis.volume.Compartmentalization" id="arivis.analysis.volume.Compartmentalization1" name="Compartments">

<inputs>[{"ObjectTags":[{"v":1,"id":"arivis.analysis.segmentation.RegionGrowing1.0.0"},{"v":1,"id":"arivis.analysis.volume.FeatureFilter1.0.0"}]}]</inputs>

<outputs>[{"ObjectTags":[{"v":1,"id":"arivis.analysis.volume.Compartmentalization1.0.0","p":null,"n":"Compartments","a":true,"cm":0,"c":"Cyan"},{"v":1,"id":"arivis.analysis.volume.Compartmentalization1.0.1","p":null,"n":"Particles Filter in

Cell_shape","a":true,"cm":4,"c":"Cyan"},{"v":1,"id":"arivis.analysis.volume.Compartmentalization1.0.2","p":
null,"n":"Cell_shape with Particles Filter","a":false,"cm":4,"c":"Cyan"}]}]</outputs>

<settings>{"NodeLevels":[1,2],"NodeSettings":[{"ProvideAsFilteredOutputForParent":false,"FilteredOutputF
orParentName":" with
Cell_shape","Modes":3,"Overlap":0.0,"MaximumDistance":0.0,"ProvideAsOutput":false,"OutputName":"Ce
ll_shape","ProvideOverlapAsFeature":false,"ProvideDistanceAsFeature":false,"ProvideModeTags":false},{"P
rovideAsFilteredOutputForParent":false,"FilteredOutputForParentName":"Cell_shape with Particles
Filter","Modes":3,"Overlap":0.15,"MaximumDistance":10.0,"ProvideAsOutput":true,"OutputName":"Particl
es Filter in
Cell_shape","ProvideOverlapAsFeature":true,"ProvideDistanceAsFeature":false,"ProvideModeTags":true}]}
</settings>

</operation>

<operation type-id="arivis.analysis.store.StoreObjects" id="arivis.analysis.store.StoreObjects1">

<inputs>[{"ObjectTags":[{"v":1,"id":"arivis.analysis.segmentation.CellposeSegmentationOperation1.0.0"},{"
v":1,"id":"arivis.analysis.volume.FeatureFilter1.0.0"},{"v":1,"id":"arivis.analysis.segmentation.RegionGrowin
g1.0.0"},{"v":1,"id":"arivis.analysis.volume.Compartmentalization1.0.1"}]}]</inputs>

<settings>{"NamePattern":"$n","TagPattern":"$n","TagsToAdd":null,"TagsToDelete":["Compartments","pa
rticles"],"PreserveAllTags":false,"MeshSegments":false,"KeepOnUndo":false}</settings>

</operation>

<operation type-id="arivis.analysis.endOfPipeline" id="arivis.analysis.endOfPipeline1">

<settings />

</operation>

</operations>

<layout>

<entry
name="arivis.analysis.store.StoreObjects1">["arivis.analysis.volume.Compartmentalization1"]</entry>

<entry
name="arivis.analysis.segmentation.RegionGrowing1">{"IntensityThreshold":{"pixeltype":"UInt8","minimu
m":"3","maximum":"255"}}</entry>

<entry name="_viewpartstates">arivis.analysis.selection1=advanced

</entry>

</layout>

<features><![CDATA[{"Version":"3.1","Features":[{"FactoryId":"arivis.objectstore.features.stored","Id":"19
e7c4ac-6857-482b-bd54-a9fb39c46350","Name":"Particles Filter in Cell_shape
overlap","Parameter":"{\"Identifier\":\"19e7c4ac-6857-482b-bd54-a9fb39c46350\",\"Name\":\"Particles

Filter in Cell_shape

overlap\",\"Values\":[{\"Name\":\"Overlap\",\"Unit\":\"Unitless\",\"DataType\":1},{\"Name\":\"Id\",\"Unit\":null,\"DataType\":10}]}"}]}]]></features>

</pipeline>
